# Iron nutrition and COVID-19 among Nigerian healthcare workers

**DOI:** 10.1093/emph/eoae034

**Published:** 2024-12-20

**Authors:** Katherine Wander, Olayinka O Ogunleye, Evelyn N Nwagu, Uche S Unigwe, Amelia N Odo, Chinedu M Chukwubike, Sunday A Omilabu, Olumuyiwa B Salu, Bukola S Owolabi, Bodunrin I Osikomaiya, Samuel O Ebede, Abimbola Bowale, Abimbola O Olaitan, Christopher U Chukwu, Chibuzo O Ndiokwelu, Chioma Edu-Alamba, Constance Azubuike, Oluwasegun A Odubiyi, Yusuf A Hassan, Nifemi Oloniniyi, Akinrinlola Muyiwa Kelvin, Raheem Rashidat Abiola, Amina Saliu, Ololade O Fadipe, Roosevelt A Anyanwu, Mercy R Orenolu, Maryam A Abdullah, Onyinye D Ishaya, Chinenye J Agulefo, Iorhen E Akase, Megan E Gauck, Zifan Huang, Mei-Hsiu Chen, Titilayo A Okoror, Masako Fujita

**Affiliations:** Department of Anthropology, Binghamton University, Binghamton, NY, USA; Department of Medicine, Lagos State University Teaching Hospital, Ikeja, Lagos, Nigeria; Department of Pharmacology, Therapeutics and Toxicology, Lagos State University College of Medicine, Ikeja, Lagos, Nigeria; Department of Human Kinetics and Health Education, University of Nigeria, Nsukka, Enugu, Nigeria; Department of Medicine, University of Nigeria Teaching Hospital, Ituku-Ozalla, Enugu, Nigeria; Department of Human Kinetics and Health Education, University of Nigeria, Nsukka, Enugu, Nigeria; Molecular Virology Unit, Department of Microbiology University of Nigeria Teaching Hospital, Ituku-Ozalla, Enugu, Nigeria; Centre for Human and Zoonotic Virology, College of Medicine, University of Lagos, Idi-Araba, Lagos, Nigeria; Department of Medical Microbiology and Parasitology, College of Medicine, University of Lagos, Idi-Araba, Lagos, Nigeria; Centre for Human and Zoonotic Virology, College of Medicine, University of Lagos, Idi-Araba, Lagos, Nigeria; Department of Medical Microbiology and Parasitology, College of Medicine, University of Lagos, Idi-Araba, Lagos, Nigeria; Department of Surgery, Lagos University Teaching Hospital, Idi-Araba, Mushin, Lagos, Nigeria; Lagos State Blood Transfusion Service, Lagos, Nigeria; Department of Haematology, Lagos State University Teaching Hospital, Ikeja, Lagos, Nigeria; Department of Medical Microbiology, University of Nigeria Teaching Hospital, Ituku-Ozalla, Enugu, Nigeria; Lagos State Civil Service, Lagos, Nigeria; Department of Medicine, University of Nigeria Teaching Hospital, Ituku-Ozalla, Enugu, Nigeria; Olabisi Onabanjo University Teaching Hospital, Sagamu, Ogun, Nigeria; Family Medicine Department, University of Nigeria Teaching Hospital, Ituku-Ozalla, Enugu, Nigeria; Department of Paediatrics, University of Nigeria Teaching Hospital, Ituku-Ozalla, Enugu, Nigeria; Molecular Virology Unit, Department of Microbiology University of Nigeria Teaching Hospital, Ituku-Ozalla, Enugu, Nigeria; Molecular Virology Unit, Department of Microbiology University of Nigeria Teaching Hospital, Ituku-Ozalla, Enugu, Nigeria; Lagos State University Teaching Hospital, Ikeja, Lagos, Nigeria; Medical Emergency Unit, Lagos State University Teaching Hospital, Ikeja, Lagos, Nigeria; Mainland Hospital, Yaba, Lagos, Nigeria; Mainland Hospital, Yaba, Lagos, Nigeria; Lagos State Health Service Commission, Lagos, Nigeria; Lagos State Health Service Commission, Lagos, Nigeria; Medical Emergency Unit, Lagos State University Teaching Hospital, Ikeja, Lagos, Nigeria; Centre for Human and Zoonotic Virology, College of Medicine, University of Lagos, Idi-Araba, Lagos, Nigeria; Centre for Human and Zoonotic Virology, College of Medicine, University of Lagos, Idi-Araba, Lagos, Nigeria; Centre for Human and Zoonotic Virology, College of Medicine, University of Lagos, Idi-Araba, Lagos, Nigeria; Usmanu Danfodiyo University Teaching Hospital, Garba Nadama, Sokoto, Nigeria; Centre for Human and Zoonotic Virology, College of Medicine, University of Lagos, Idi-Araba, Lagos, Nigeria; Lagos University Teaching Hospital, Idi-Araba, Mushin, Lagos, Nigeria; College of Medicine, University of Lagos (CMUL), Idi-Araba, Lagos, Nigeria; Department of Anthropology, Binghamton University, Binghamton, NY, USA; Department of Mathematics and Statistics, Binghamton University, Binghamton, NY, USA; Department of Mathematics and Statistics, Binghamton University, Binghamton, NY, USA; Department of Africana Studies, Binghamton University, Binghamton, NY, USA; Department of Anthropology, Michigan State University, East Lansing, MI, USA

**Keywords:** optimal iron hypothesis, emerging infectious disease, evolutionary epidemiology, SARS-CoV-2

## Abstract

**Background and objectives:**

The optimal iron hypothesis (OIH) posits that risk for infection is lowest at a mild level of iron deficiency. The extent to which this protection results from arms race dynamics in the evolution of iron acquisition and sequestration mechanisms is unclear. We evaluated the OIH with regard to severe acute respiratory syndrome coronavirus 2 (SARS-CoV-2), an emerging infectious agent.

**Methodology:**

We tested 304 healthcare workers at baseline for iron deficiency (zinc protoporphyrin:heme), anemia (hemoglobin), and SARS-CoV-2 (salivary PCR), and followed them for ~3 months with biweekly SARS-CoV-2 tests. We fit logistic regression models based on Akaike Information Criterion.

**Results:**

Adequate data were available for 199 participants. Iron replete (OR: 2.87, 95% CI: 0.85, 9.75) and anemia (OR: 2.48; 95% CI: 0.82, 7.85) were associated with higher risk for SARS-CoV-2 infection after control for covariates. Logistic regression and Cox proportional hazards models of the SARS-CoV-2 outcome were similar. Anemia (OR: 1.81; 95% CI: 0.88, 3.71) was associated with respiratory symptoms regardless of SARS-CoV-2 infection.

**Conclusions and implications:**

These findings provide partial support for the OIH: SARS-CoV-2 infection risk was elevated at the high end of the range of iron availability; however, the elevated risk among those with anemia was not, as expected, specific to severe iron deficiency. Narrowly, for COVID-19 epidemiology, these findings accord with evidence that SARS-CoV-2’s ability to establish infection is enhanced by access to iron. More broadly, these findings suggest that the OIH does not hinge on a long history of evolutionary arms race dynamics in access to host iron.

## BACKGROUND AND OBJECTIVES

Iron nutrition can have multiple, complex effects on infectious disease risk. Both immune cells and infectious agents require iron to support their function. Because iron that is available to host cells is also available to infectious agents, it may be that risk for at least some infectious diseases is decreased by iron intake that is inadequate to meet the body’s overall iron needs. In other words, mild iron deficiency, relative to either the iron-replete state or severe iron deficiency, may be optimal, at least for infectious disease risk [1–4]. We have found support for this hypothesis in multiple settings in sub-Saharan Africa [1, 5, 6]. Here, we test whether iron deficiency affects risk for infection with severe acute respiratory syndrome coronavirus 2 (SARS-CoV-2), the causal agent of coronavirus disease 2019 (COVID-19), among healthcare workers (HCW) in Nigeria in 2021–22.

Testing the optimal iron hypothesis (OIH) with regard to SARS-CoV-2 risk is important to understanding the evolutionary grounding of this hypothesis. Iron is a limiting resource for many infectious agents. Viruses rely on host iron to efficiently replicate within infected cells, and employ multiple and overlapping mechanisms to access host iron [7, 8]. While evidence of evolutionary arms race dynamics between an infectious agent and host is most apparent for bacterial pathogens—whose abilities to extract host iron, even in the face of multiple host mechanisms to sequester it, provide clear evidence of the iterative nature of this arms race [9–11]—it is likely that these dynamics are also at play for intracellular access to iron for many viruses [7, 12, 13]. It is against the background of these evolutionary arms race dynamics that we have posited that, as infectious agents evolve mechanisms to subvert iron sequestration and extract host iron, nutritional iron deficiency may hinder infections and lower infectious disease risk or severity. SARS-CoV-2, as an emerging infectious agent (or emerging infectious disease, EID), may be poorly adapted to humans’ iron sequestration and withholding mechanisms. If iron sequestration is effective in limiting the iron available to support SARS-CoV-2 replication, there is unlikely to be any hazard to the iron-replete state (or conversely, any benefit to the iron-deficient state). Instead, those who are iron replete may have the best delivery of iron to immune defense and so the lowest risk for SARS-CoV-2 infection.

However, some evidence suggests that iron is particularly salient to risk for infection with SARS-CoV-2. SARS-CoV-2 attacks hemoglobin, freeing iron from porphyrins and increasing free iron [14, 15]. This free iron plays a role in COVID-19’s pathogenesis [14], and a positive association between iron availability and disease severity has been reported among COVID-19 patients [16]. As with some other viruses that affect iron homeostasis, however, it remains unclear the extent to which these effects enhance SARS-CoV-2’s ability to establish an infection or persist in the host (or whether these effects are byproducts of viral proliferation) [7]. Lower risk for SARS-CoV-2 among those with blood type O [17–19] has been suggested to be attributable to lower circulating iron availability [14], but this is far from definitive evidence that iron is central to SARS-CoV-2’s ability to establish an infection. Initial investigations of hemochromatosis as a risk factor for SARS-CoV-2 infection have mixed results, but overall present limited evidence that one disease-associated allele (and resultant higher plasma iron) may increase risk for SARS-CoV-2 infection or a more severe course of COVID-19 [20, 21].

Thus, overall, there is reason to expect that iron deficiency increases risk for SARS-CoV-2 infection *and* reason to expect that it reduces risk: If SARS-CoV-2 is poorly adapted to humans’ many mechanisms of iron withholding and sequestration, then iron-replete and iron deficient hosts are unlikely to differ in the availability of iron for SARS-CoV-2, and iron deficient hosts may be at higher risk for infection due to weakened immune defense. On the other hand, SARS-CoV-2 may need free iron, generated by attacking host hemoglobin, to enhance its ability to establish an infection and efficiently replicate in host cells, in which case an iron-replete host may have a higher risk for infection. We assessed the impact of iron nutrition on risk for SARS-CoV-2 infection. Our goals in this project were two-fold: adding nuance to our understanding of the OIH and its place in the broader study of the evolutionary dynamics of humans and pathogens, and expanding our understanding of COVID-19 epidemiology.

## METHODOLOGY

### Participants and setting

We collected data from three hospitals in Lagos state and one hospital in Enugu state in Nigeria. Participants were HCW in units most likely to treat COVID-19 patients (dedicated COVID-19 wards and medical emergency wards). We invited all HCW, including both providers (physicians and nurses) and support staff, from selected wards to participate, until target sample sizes (200 in Lagos, 100 in Enugu) were met. Hospital employees who held administrative roles that did not involve contact with patients were not included. Participation included an initial visit, at which hypothesized risk and protective factors were characterized and the first COVID-19 test was performed, followed by weekly surveys for symptoms and biweekly COVID-19 testing (PCR positivity persists for ~2 weeks, often longer [22, 23], so while this testing interval did not allow us to pinpoint when participants became positive, it was unlikely to miss many cases).

We obtained written informed consent from all participants. The Institutional Review Boards of Lagos State University Teaching Hospital (LASUTH), the Lagos University Teaching Hospital, and the University of Nigeria Teaching Hospital (UNTH) provided ethical review and oversight. Binghamton University’s IRB relied on the findings and oversight of LASUTH and UNTH. All procedures were in accordance with the ethical standards of the review boards and with the 1964 Helsinki Declaration and its later amendments.

### Survey instruments

The initial survey instrument asked all participants to describe basic personal characteristics (e.g. date of birth), household size and composition information, their role within the hospital (physician/nurse/other), their highest degree earned, COVID-19 vaccination status, past positive COVID-19 tests, and past diagnoses with diabetes or other chronic disease. Follow-up weekly surveys asked participants to report any of a list of infectious disease symptoms, which included COVID-19-specific symptoms (e.g. loss of taste and smell) and respiratory infectious diseases more generally (e.g. fever, cough); follow-up surveys also asked participants to report any new COVID-19 vaccination received or positive COVID-19 test from a source outside the study.

### Anthropometry

At the initial data collection, we characterized weight and height with the hospitals’ standard equipment.

### Hematology

A trained phlebotomist collected venous blood at the initial data collection. Specimens were transported on ice to the Hematology Laboratory at the Mainland Hospital (Lagos) or the Molecular Virology Laboratory at UNTH (Enugu), where we assessed whole blood specimens for hemoglobin (Hb) using a HemoCue Hb 301 hemoglobinometer; zinc protoporphyrin to heme ratio (ZPP:H) using a hematofluorometer (ProtoFluor-Z, Helena Laboratories); and glycated hemoglobin (HbA_1C_) using an Infopia Clover A1c analyzer in Lagos and a SimmplexTAS analyzer in Enugu.

### Virology

Participants provided saliva specimens in sterile containers at the initial data collection and biweekly for 3 months. ~1–2 ml of whole saliva was transported on ice to the Department of Medical Microbiology Research Laboratory, at the College of Medicine of the University of Lagos (Lagos) or the Molecular Virology Laboratory at UNTH (Enugu) and stored at −60°C until analysis. Following the manufacturer’s instructions, we extracted viral nucleic acid from inactivated specimens using a small spin column RNA extraction kit (Qiagen, Maryland, USA). We amplified and reverse-transcribed purified ribonucleic acid (RNA) into complementary DNA using the GeneFinder COVID-19 Plus RealAmp RT-PCR test kit. This kit employs qRT-PCR for the qualitative identification of the SARS-CoV-2 RdRp, N, and E genes. We considered results valid if internal control and cycle threshold values were within the kit manufacturer’s acceptable ranges.

### Statistical analyses

We parameterized biomarker variables as follows: diabetes, HbA1c ≥ 6.5%; anemia, Hb < 13.0 mg/dl for males and Hb < 12.0 mg/dl for females [24]; iron deficiency, ZPP:H ≥ 70 μmol/mol [25]. Because consensus around a ZPP:H definition for iron deficiency is lacking, with published cutpoints ranging from 40 to 80 μmol/mol [1, 25–29], and in recognition of the arbitrary nature of cutpoint-based definitions, we also trialed cutpoints of ZPP:H ≥ 80 μmol/mol for iron deficiency and ZPP:H < 40 μmol/mol for iron replete [1]. We treated no response as missing information for survey items, with the exception of previous diagnoses, weekly symptoms, COVID-19 vaccination, or co-resident household members; for these variables, we assumed blank responses indicated “no” or “0” (as “no” or “0” were rarely recorded responses).

We excluded participants from analyses if they missed more than two scheduled PCR tests. We then considered any positive SARS-CoV-2 PCR test the primary outcome of interest. Other outcomes of interest include symptomatic COVID-19 (a positive PCR test combined with cough, sore throat, fever, shortness of breath, and/or loss of taste/smell), and symptomatic respiratory infection (reported cough, sore throat, shortness of breath, fever, and/or loss of taste/smell, regardless of SARS-CoV-2 PCR test results).

We fit logistic regression models for each outcome of interest, using the Akaike Information Criterion (AIC) to select the best-fit model among nested models. Predictors of interest included iron deficiency or replete (by ZPP:H) and anemia (by Hb), as well as the interaction between them (the OIH predicts an interaction between iron deficiency and anemia such that a protective effect of iron deficiency is limited to those without anemia, or mild to moderate iron deficiency). We considered COVID-19 vaccination, diabetes, overweight (body mass index, BMI, ≥ 25) and/or obesity (BMI ≥ 30), hospital role, study site, and household size (number of reported co-resident children and adults) as potential confounding variables.

## RESULTS

### Descriptive analyses

Three hundred four participants initially enrolled in the study; adequate data for analysis was available for 199. A total of 105 individuals were excluded from the analysis set: 5 individuals were assigned a study ID and then declined to further participate; an additional 71 were excluded for missing 3 or more PCR tests; an additional 23 were excluded for missing BMI; an additional 5 individuals were excluded for missing HbA_1C_; and an additional 1 was excluded for missing hospital role. The 199 participants included in analyses were not markedly different in characteristics from the initial 304 participants (Table 1).

**Table 1.** Sample characteristics.

	All enrolled (*n* = 304)		All analyzed (*n* = 199)	
	Number	Percentage	Number	Percentage
Sex				
Female	170	56	111	56
Male	129	42	88	44
Missing data	5	2	0	0
Hospital site				
Enugu	100	33	80	40
Lagos	204	67	119	60
Hospital role				
Physician	65	21	55	28
Non-Physician	233	77	144	72
Missing data	6	2	0	0
COVID-19 vaccination status				
Yes	212	70	137	69
No	92	30	62	31
Type 2 diabetes				
Yes	37	12	22	11
No	249	82	177	89
Missing data	18	6	0	0
COVID-19 PCR test results				
Any Positive	26	9	22	11
Never Positive	202	66	177	89
Missing data	76	25	0	0
Respiratory symptoms*				
Yes	101	33	76	38
Never	203	67	123	62
Anemia				
Yes	99	32	65	33
No	191	63	134	67
Missing data	14	5	0	0
Iron deficiency (ZPP:H ≥ 70 μmol/mol heme)				
Yes	58	19	32	16
No	232	76	167	84
Missing data	14	5	0	0
Iron replete (ZPP:H < 40 μmol/mol heme)				
Yes	55	18	41	21
No	235	77	158	79
Missing data	14	5	0	0
	Mean | Std Dev	Range	Mean | Std Dev	Range
Age in years	37.77 | 9.15	21-61	37.48 | 9.55	21–61
BMI	27.27 | 4.76	17.85-42.22	27.11 | 4.52	17.85–42.22
Household size (<18 years old)	1.44 | 1.65	0-10	1.48 | 1.63	0–10
Household size (≥18 years old)	2.66 | 2.18	1-19	2.85 | 2.21	1–19

*Respiratory symptoms include reported fever, cough, shortness of breath, sore throat, and/or loss of smell or taste in weekly symptom questionnaires.

Both iron deficiency (16%) and anemia (33%) were common. 11% of participants tested positive at least once for SARS-CoV-2 during the data collection period. Symptomatic COVID-19 was uncommon among our participants, likely due to high rates of vaccination (most vaccinated participants reported receiving the Oxford/AstraZeneca vaccine, which has better efficacy for preventing severe or symptomatic disease than infection [30–32]).

The majority of positive test results occurred early in the evaluation period, including 41% of observed cases on the first test date. As such, we relied primarily on logistic regression analyses to test the hypothesized relationships between iron deficiency, anemia, and SARS-CoV-2 infection.

### SARS-CoV-2 infection

Comparisons of models by AIC did not support the inclusion of iron deficiency variables (ZPP:H ≥ 70 or ≥80 μmol/mol) in models of the SARS-CoV-2 outcome, alone or in interaction with anemia. Model selection by AIC did support inclusion of iron replete (ZPP:H < 40 μmol/mol definition) in the final model, as well as anemia, age (in ~10-year increments of 20–29, 30–39, 40–49, and 50+), lean BMI (BMI < 25), household size including only adults (stratified as small or <4, medium or 4–5, and large or >5), and study site (Table 2). SARS-CoV-2 infection occurred more often among iron-replete HCW (OR: 2.87, 95% CI: 0.85, 9.75; Table 3) and those with anemia (OR: 2.48; 95% CI: 0.82, 7.85). SARS-CoV-2 infection also occurred more often among older participants, was less common among those with BMI < 25, and occurred more often among those in the Enugu study site.

**Table 2.** Model selection for PCR-positive SARS-CoV-2 infection.

	Model 1	Model 2	Model 3	Model 4	Model 5	Model 6	Model 7	Final model
	Anemia	Anemia	Anemia	Anemia	Anemia	Anemia	Anemia	Anemia
	Site	Site	Site	Site	Site	Site	Site	Site
	Age*	Age*	Age*	Age*	Age*	Age*	Age*	Age*
	Household size (adults**)	Household size (adults**)	Household size (adults**)	Household size (adults**)	Household size (adults**)	Household size (adults**)	Household size (adults**)	Household size (adults**)
	Hospital role	Hospital role	Hospital role	Hospital role	Hospital role	Hospital role	Hospital role	
	Iron replete	Iron replete	Iron replete	Iron replete	Iron replete	Iron replete		Iron replete
	BMI < 25	BMI < 25	BMI < 25	BMI < 25	BMI < 25		BMI < 25	BMI < 25
	Vaccine	Vaccine	Vaccine	Vaccine				
	Sex	Sex	Sex					
	Household size (children***)	Household size (children***)						
	Diabetes by HbA_1C_							
AIC	129.46	127.47	124.35	123.25	121.34	121.93	121.40	120.08

*Age in 10-year increments, with 2 participants aged 60 and 61 years combined with the 50–59 group;

**age ≥ 18 years;

***age < 18 years.

**Table 3.** Final model of PCR-positive SARS-CoV-2 infection.

Variable	Regression coefficient	Coefficient 95% CI		Odds ratio	Odds ratio 95% CI		*P*-value
Intercept	−4.43	−6.04	−2.83				
Anemia	0.91	−0.21	2.03	2.48	0.82	7.85	.11
Iron replete	1.05	−0.14	2.25	2.87	0.85	9.57	.08
Low BMI	−1.08	−2.49	0.34	0.34	0.07	1.26	.14
Age*	0.54	-0.02	1.10	1.71	1.00	3.08	.06
Site (Enugu)	1.22	0.02	2.42	3.38	1.05	11.93	.05
Household size—adults (small)	Ref						
Household size—adults (medium)	1.37	0.21	2.53	3.95	1.22	12.82	.02
Household size—adults (large)	0.96	-0.52	2.43	2.61	0.54	10.94	.20

*Age in 10-year increments, with 2 participants aged 60 and 61 years combined with the 50–59 group.

We also estimated a Cox proportional hazards (CPH) regression model with the same predictor variables. CPH models have the advantage of accounting for time at risk; however, the preponderance of events early in the monitoring period limits our ability to rely on these models. An additional 20 participants (19 negative and 1 positive) were excluded from these models due to missing information on exact dates for at least one PCR test. This model showed similar patterns to the logistic regression model (Supplementary Table S1).

### Respiratory infection

Comparisons of models by AIC did not support inclusion of any iron nutrition variables in models of the symptoms of respiratory infectious disease outcome (fever, cough, sore throat, shortness of breath, and/or loss of taste or smell). Using AIC, the final model for respiratory symptoms included anemia, age (in 10-year increments), sex, household size (adults and children, continuous), lean BMI, and study site (Table 4). Like SARS-CoV-2 infection, respiratory infectious disease symptoms were more common among HCW with anemia (OR: 1.81; 95% CI: 0.88, 3.71), although this pattern was more pronounced for SARS-CoV-2 infection than for respiratory symptoms (Table 5). Unlike SARS-CoV-2 infection, symptoms of respiratory infection declined with age (OR: 0.68, 95% CI: 0.48, 0.96). Respiratory infection symptoms also increased with household size and were more common in Enugu.

**Table 4.** Model selection for respiratory infectious disease symptoms.

	Model 1	Model 2	Model 3	Model 4	Model 5	Final model
	Anemia	Anemia	Anemia	Anemia	Anemia	Anemia
	Site	Site	Site	Site	Site	Site
	Age*	Age*	Age*	Age*	Age*	Age*
	Household size	Household size	Household size	Household size	Household size	Household size
	Hospital role	Hospital role	Hospital role	Hospital role	Hospital role	
	Iron replete	Iron replete	Iron replete	Iron replete		
	BMI < 25	BMI < 25	BMI < 25			
	Sex	Sex		Sex	Sex	Sex
	Diabetes by HbA_1c_					
AIC	253.8	252.1	254.5	252.1	250.5	248.5

*Age in 10-year increments, with 2 participants aged 60 and 61 years combined with the 50-59 group;

**age ≥ 18 years;

***age < 18 years.

**Table 5.** Final model of respiratory infectious disease symptoms.

Variable	Regression coefficient	Coefficient 95% confidence interval		Odds ratio	Odds ratio 95% confidence interval		*P*-value
Intercept	−0.68	−1.41	0.05				.07
Anemia	0.59	−0.12	1.31	1.81	0.88	3.71	.10
Age*	−0.38	-0.73	−0.04	0.68	0.48	0.96	.03
Male sex	−0.66	−1.33	0.02	0.52	0.26	1.01	.06
Site (Enugu)	0.75	0.00	1.50	2.11	1.00	4.50	.05
Total household size	0.10	−0.02	0.22	1.10	0.98	1.25	.11

*Age in 10-year increments, with 2 participants aged 60 and 61 years combined with the 50–59 group;

**age ≥ 18 years;

***age < 18 years.

Thus, in sum, our models suggest that iron replete was positively associated with SARS-CoV-2 infection and anemia was positively associated with SARS-CoV-2 infection and symptoms of respiratory infection more generally. This is partially consistent with the OIH—in that, it suggests SARS-CoV-2 infection risk is higher among those with more iron available—although we would also have expected that association to extend to respiratory infections more broadly (as captured by fever, cough, shortness of breath, and sore throat). The positive associations between anemia and both SARS-CoV-2 infection and respiratory infection are also consistent with our expectations of higher risk for infectious disease when nutritional strain is enough to compromise immunity. However, iron status did not interact with anemia in the way that we predicted (a protective effect of mild/moderate iron deficiency; i.e., a protective effect of iron deficiency only among those who were not anemic), and so we cannot attribute the positive association between anemia and COVID-19 or infectious symptoms to severe iron deficiency alone.

Broadly, our results are consistent with the hypothesis that abundant host iron nutrition increases risk for SARS-CoV-2 infection.

## CONCLUSIONS AND IMPLICATIONS

### The optimal iron hypothesis

The impact of iron nutrition on infectious disease risk results from multiple complex interactions between immunity, nutrition, and infectious agents that are almost certainly simplified by the OIH. Iron nutrition is dynamic, and not only affects, but is affected by, infectious disease processes: Infectious agents in the gastrointestinal tract can cause blood loss or compete with the host for dietary iron [33–37]. Others, including SARS-CoV-2, cause the destruction of erythrocytes or hemoglobin, disrupting the use of iron for oxygen transportation and increasing free iron in a way that is damaging to host tissues [35, 36]. The immune response to infection likely increases iron demands, while also severely limiting iron absorption and sequestering iron away from cellular use, redistributing it to more secure compartments (e.g. bound to ferritin within macrophages), which some infectious agents have evolved to exploit [9, 38–43]. The complexity of these dynamics across infectious agents, environments, and hosts tremendously complicates investigations of the OIH. Understanding the conditions in which iron deficiency is and is not protective will be important in further understanding the evolutionary dynamics of human infectious agents, and how these affect iron nutrition and disease vulnerability.

One interesting question within investigations of the OIH regards the role of evolutionary arms race dynamics. We have argued here and elsewhere [1, 5, 6] that protective effects of iron deficiency against infection arise at least in part from these arms race evolutionary dynamics between humans and our infectious agents: infectious agents’ iron acquisition mechanisms have an evolutionary advantage in their faster generation times, and many have overcome multiple iron defense and sequestration mechanisms, and so even in the presence of complex feedback pathways between iron nutrition and infection, across a broad range of infectious agents, environments, and hosts, we expect restricted iron intake and absorption to reduce risk for infection. However, empirical evidence to evaluate the question, “Do evolutionary arms race dynamics create conditions in which iron deficiency may constitute a nutritional adaptation to infectious disease”? is limited. We posit that EID can address this question, as emerging infectious agents are often poorly adapted to the human host and lack a long history of evolutionary arms race dynamics with humans. Here, by assessing iron nutrition and risk for an EID (COVID-19, or SARS-CoV-2 infection), we begin to assess whether the OIH is indeed contingent on a long evolutionary arms race for host iron.

Overall, our analyses suggest that the OIH is partially supported for at least one EID: the iron-replete state seems to increase risk for SARS-CoV-2 infection. This counters our assertion that arms race dynamics contributed to the selection of iron deficiency as a nutritional adaptation to infectious disease. We also found that anemia seems to increase risk for SARS-CoV-2 infection, but that this risk is not limited to iron deficiency anemia. This provides much more limited evidence for the predicted protective effect of mild iron deficiency (relative to either the replete or severe deficiency states) than we have previously documented [5, 6], but does counter the expectation that the OIH is relevant primarily or exclusively for infectious agents that have evolved to subvert or exploit mechanisms of iron withholding. While this is only one EID, and our findings are not conclusive on their own, they do suggest that a long history of arms race dynamics is not necessary for nutritional adaptations that restrict iron availability to infectious agents to have a protective effect against infection. Alternatively, SARS-CoV-2, through arms race dynamics with other mammalian host organisms [44], may have been pre-adapted to subvert human iron sequestration mechanisms, providing a poor test of the role of arms race dynamics in the OIH.

We found an elevated risk for SARS-CoV-2 infection in the most iron-replete state, rather than a lower risk in the state of frank iron deficiency. This may be explained by differences across studies in iron deficiency biomarkers and cutpoints (e.g. our cutpoint for iron-replete represents the low end of the range of values used to define iron deficiency); it may also arise from real differences across samples, settings, or infectious agents in associations between iron nutrition and infection risk. Tradeoffs in iron nutrition, between iron available to host cells and infectious agents, may exist in some settings without pushing optimal iron into the range of deficiency. Nonetheless, our results clearly suggest replete iron nutrition (as indicated by ZPP:H, which is specific to iron nutrition but lacks consensus around cutpoints [25, 27–29, 45]) is associated with elevated risk for SARS-CoV-2 infection. Our results may also indicate that anemia (as indicated by Hb, which is affected by iron nutrition as well as multiple other factors affecting erythropoiesis [46, 47]) is associated with elevated risk for SARS-CoV-2 infection. This may suggest, as the OIH predicts, higher risk at both ends of the range of iron availability. However, the effect of anemia among our participants was not limited to iron deficiency anemia, limiting the interpretability of this result with regard to the OIH.

In contrast to our findings for SARS-CoV-2 infection, we found little support for the OIH with regard to respiratory infections in general—iron nutrition was unassociated with disease risk. This was unexpected, in light of previous work. However, data in support of the OIH to date come largely from children [1, 5, 48] and postpartum mothers [6], including those living through sustained droughts [1, 6]. The divergent findings in this study may point to participants’ underlying capacity for immune defense—which the OIH posits declines with limited iron availability—as an important determinant of optimal iron, such that limited iron nutrition is more likely to be protective when the immune defense is compromised (e.g. due to immaturity).

### SARS-CoV-2 epidemiology

We observed some expected patterns in risk for SARS-CoV-2 infection: a lean BMI (<25) was protective, and risk increased with age. Notably, we observed an increasing risk for SARS-CoV-2 infection with age among non-elderly HCW (our oldest participant was 61 years of age, younger than many definitions of “elderly” employed in COVID-19 public health policy [49, 50]). This finding suggests that public health and prevention programs may benefit from incorporating more nuance into age-based recommendations. Overall, the consistency of our models with established patterns in COVID-19 risk [51, 52] gives us confidence in our novel finding of elevated risk among iron-replete and anemic participants.

Elevated risk among those who are iron replete is consistent with previously published suggestions that the SARS-CoV-2 virus benefits from higher iron availability, such as higher risk among carriers of at least one hemochromatosis-associated allele. This suggests that SARS-CoV-2’s ability to establish an infection is enhanced by its attack on hemoglobin and the resultant increase in free iron. Other authors have suggested iron chelation as a potential therapeutic for COVID-19 [15, 53]; future research may consider not only whether iron chelation alleviates the adverse effects of hemoglobin destruction and excess free iron, but whether it impairs SARS-CoV-2 replication in the host.

### Limitations

The models in Tables 3 and 5 are supported by the assessment of fit using an information theoretic approach. Nonetheless, caution in interpreting these results is merited, given the wide confidence intervals for iron replete and anemia.

Most cases of SARS-CoV-2 infection in our data occurred early in the monitoring period (including some at the initial visit), limiting our ability to capitalize on the longitudinal nature of these data in modeling; as a result, although our approach (logistic regression for any positive test during monitoring) was statistically robust, our findings are vulnerable to misattribution of cause and effect.

Due to the complex interactions between iron nutrition and infection, previous tests of the OIH have found that cross-sectional and longitudinal study designs can lead to disparate conclusions [2, 5], with cross-sectional assessments potentially capturing the effects of infection and inflammation on risk for iron deficiency anemia, rather than effects of iron nutrition on risk for infection. This is likely a concern with SARS-CoV-2 infection, as well, as it can have hemolytic effects [14, 16, 53]. Here, we have longitudinal data, but since the majority of infections happened early in the monitoring period, we have essentially collapsed the time component of our data to use logistic regression models. Thus, it could be that the positive association between anemia and SARS-CoV-2 infection that we observed actually reflects SARS-CoV-2 infection increasing risk for anemia, not the converse. However, this explanation seems unlikely, given the low rate of otherwise symptomatic infections we observed; further, reverse causation is not a plausible explanation for the positive association between SARS-CoV-2 infection and the iron-replete state that we also report.

We relied on a biomarker of iron deficiency, ZPP:H, that is more robust to inflammation than many others (e.g. ferritin or hepcidin [54]); nonetheless, some studies have reported elevated ZPP:H in the presence of inflammation [54, 55]. We do not have information about inflammation and so have a limited ability to discriminate between nutritional iron deficiency and iron withholding as determinants of ZPP:H. However, it is unlikely that elevated ZPP:H due to inflammation at the outset of data collection would explain the positive association we observed between iron-replete (low ZPP:H) and SARS-CoV-2 infection. Similarly, we do not have information about hemoglobinopathies, which may elevate ZPP:H [54]; it is thus possible that a protective effect of hemoglobinopathy-associated alleles against SARS-CoV-2 infection contributed to our findings.

Missingness was high in these data, mostly due to poor attendance for COVID-19 testing. Missing follow-up visits are likely attributable to high workloads among participating HCW during the periods of data collection in both Lagos and Enugu; if those who were excluded due to missed tests were also those who were busiest with patient care, they may have been particularly likely to be infected with SARS-CoV-2, resulting in under-ascertainment of cases. This is unlikely, however, to have falsely produced any of the associations between SARS-CoV-2 and iron deficiency or anemia that we observed.

PCR testing is vulnerable to false negatives, particularly in the early days of an infection [56]. There is heterogeneity in how long people remain PCR positive for SARS-CoV-2 after infection, with many remaining positive for a period of multiple weeks [57]. While we selected a 2-week testing interval to accommodate our participants’ high workloads while minimizing missed cases, it is possible that a case occurred in the period between tests that was not captured by our sampling schedule.

## CONCLUSIONS

Both replete iron nutrition and anemia may increase risk for SARS-CoV-2 infection. For biological anthropologists and others interested in adaptation to infectious disease, this finding suggests that the OIH is not contingent on a history of a long evolutionary battle between host and infectious agent over iron. For those interested in SARS-CoV-2 virology and evolution, this finding bolsters arguments that accessing host iron is a key component of the virus’s ability to establish an infection.

## Supplementary Material

eoae034_suppl_Supplementary_Material
