# Supplementary material for: Iron nutrition and COVID-19 among Nigerian healthcare workers
Source: Evol Med Public Health. 2024 Dec 20;12(1):287–97. doi: 10.1093/emph/eoae034 (PMC11697216; doi:10.1093/emph/eoae034)
Supplement: eoae034_suppl_Supplementary_Material [file eoae034_suppl_supplementary_material.pdf]

Iron nutrition and COVID-19 among Nigerian healthcare workers

Katherine Wander*[1], Olayinka O Ogunleye[2,3], Evelyn N Nwagu[4], Uche S Unigwe[5], Amelia N Odo[4], Chinedu M Chukwubike[6], Sunday A Omilabu[7,8], Olumuyiwa B Salu[7,8], Bukola S Owolabi[9], Bodunrin I Osikomaiya[10,11], Samuel O Ebede[12], Abimbola Bowale[13], Abimbola O Olaitan[14,5], Christopher U Chukwu[15], Chibuzo O Ndiokwelu[16], Chioma Edu-Alamba[6], Constance Azubuike[6], Oluwasegun A Odubiyi[17], Yusuf A Hassan[18], Nifemi Oloniniyi[19], Muyiwa Kelvin Akinrinlola[19], Rashidat Abiola Raheem[20], Amina Saliu[20], Ololade O Fadipe[18], Roosevelt A Anyanwu[7], Mercy R Orenolu[7], Maryam A Abdullah[7], Onyinye D Ishaya[21], Chinenye J Agulefo[7], Iorhen E Akase[22,23], Megan E Gauck[1], Zifan Huang[24], Mei-Hsiu Chen[24], Titilayo A Okoror[25], Masako Fujita[26]

1 Department of Anthropology, Binghamton University, Binghamton, New York, USA

2 Department of Medicine, Lagos State University Teaching Hospital, Ikeja, Lagos, Nigeria

3 Department of Pharmacology, Therapeutics and Toxicology, Lagos State University College of Medicine, Ikeja, Lagos, Nigeria

4 Department of Human Kinetics and Health Education, University of Nigeria, Nsukka, Enugu Nigeria

5 Department of Medicine, University of Nigeria Teaching Hospital, Ituku-Ozalla, Enugu, Nigeria

6 Molecular Virology Unit, Department of Microbiology UNTH, Ituku-Ozalla, Enugu, Nigeria

7 Centre for Human and Zoonotic Virology, College of Medicine, University of Lagos, Idi-Araba, Lagos, Nigeria

8 Department of Medical Microbiology and Parasitology, College of Medicine, University of Lagos, Idi-Araba, Lagos, Nigeria

9 Department of Surgery, Lagos University Teaching Hospital, Idi-Araba, Mushin, Lagos, Nigeria

10 Lagos State Blood Transfusion Service, Lagos, Nigeria

11 Department of Haematology, Lagos State University Teaching Hospital, Ikeja, Lagos, Nigeria

12 Department of Medical Microbiology, University of Nigeria Teaching Hospital, Ituku-Ozalla, Enugu, Nigeria

13 Lagos State Civil Service, Lagos, Nigeria

14 Olabisi Onabanjo University Teaching Hospital, Sagamu, Ogun, Nigeria

15 Family Medicine Department, University of Nigeria Teaching Hospital, Ituku-Ozalla, Enugu, Nigeria

16 Department of Paediatrics, University of Nigeria Teaching Hospital, Ituku-Ozalla, Enugu, Nigeria

17 Lagos State University Teaching Hospital, Ikeja, Lagos, Nigeria

18 Medical Emergency Unit, Lagos State University Teaching Hospital, Ikeja, Lagos, Nigeria

19 Mainland Hospital, Yaba, Lagos, Nigeria

20 Lagos State Health Service Commission, Lagos, Nigeria

21 Usmanu Danfodiyo University Teaching Hospital, Garba Nadama, Sokoto, Nigeria

22 Lagos University Teaching Hospital, Idi-Araba, Mushin, Lagos, Nigeria

23 College of Medicine, University of Lagos (CMUL), Idi-Araba, Lagos, Nigeria

24 Department of Mathematics and Statistics, Binghamton University, Binghamton, New York, USA

25 Department of Africana Studies, Binghamton University, Binghamton, New York, USA

26 Department of Anthropology, Michigan State University, East Lansing, Michigan, USA

*Corresponding author: katherinewander@binghamton.edu

Supplemental Information

Table S1. Cox proportional model of PCR-positive SARS-CoV-2 infection							
Variable	Regression Coefficient	Coefficient 95% CI		Hazard Ratio	Hazard Ratio 95% CI		P-value
Anemia	0.81	-0.14	1.76	2.25	0.87	5.84	0.10
Iron replete	0.59	-0.36	1.55	1.81	0.70	4.69	0.22
BMI < 25	-0.82	-2.16	0.53	0.44	0.12	1.69	0.23
Age*	0.44	-0.07	0.95	1.55	0.94	2.58	0.09
Site (Enugu)	1.01	-0.06	2.08	2.75	0.94	8.00	0.06
Household size – adults (small)	Ref						
Household size – adults (medium)	1.08	0.10	2.07	2.95	1.10	7.91	0.03
Household size – adults (large)	0.69	-0.56	1.93	1.99	0.57	6.92	0.28

*Age in 10-year increments, with 2 participants aged 60 and 61 years combined with the 50-59 group
